# Adipose-Derived Stem Cells Are an Efficient Treatment for Fistula-in-ano of Japanese Rabbit

**DOI:** 10.1155/2019/6918090

**Published:** 2019-10-22

**Authors:** Xiao Qin, Peng Wang, Yongming Huang, Yansen Li, Min Chao, Wei Wang

**Affiliations:** ^1^Department of Anorectal Surgery, Affiliated Hospital of Jining Medical University, Jining Medical University, Jining, 272029 Shandong, China; ^2^Department of Pathology, Affiliated Hospital of Jining Medical University, Jining Medical University, Jining, 272029 Shandong, China

## Abstract

Fistula-in-ano (FIA, anal fistula) treatment remains a surgical challenge for coloproctologists. Adipose tissue-derived stem cells (ADSCs) are a new frontier in the treatment of FIA. In this study, we established a FIA model of Japanese rabbit and evaluated the effect of four treatments on fistula healing: ADSC transplantation, acellular small intestinal submucosa (ASIS), noncutting seton, and PBS as negative control. High-throughput RNA sequencing was also performed to investigate the anal tissue (normal and ADSC treatment group) expression profile of mRNA. Our data showed that ADSC treatment had the shortest time to promote fistula healing compared to the other treatments, and fistula filled with new adipose tissue and muscle cells without scar. Gene Ontology (GO) analysis of RNA-seq data showed that the differential genes are enriched in system development and animal organ development. Taken together, our findings demonstrate that ADSCs rapidly promote fistula healing through differentiation, a promising stem cell therapy for FIA. The rabbit is an effective animal model for evaluating FIA therapeutic options.

## 1. Introduction

FIA is one of the most common anorectal diseases, within challenging clinical condition for both diagnosis and treatment [1], and its incidence is 9104-16645 cases per year in the EU [2] and 2000-25000 cases per year in the USA [3]. Males are affected twice as commonly as females [4]. Several surgical procedures to treat FIA have been developed in recent decades, but patients complain about discomfort and the prolonged healing period [5]. Cutting seton is a key method to treat complex anal fistula, which can protect sphincter function to some extent; however, the anal incontinence rate is still up to 34%-63%, and the postoperative pain is severe [6]. The anal fistula plug (AFP), as a sphincter-preserving technique, is simple, minimally invasive, and with a short length of stay [7], but the role of AFP in the treatment of complex cryptoglandular anal fistula is still not well established [8, 9].

The mesenchymal stem cell (MSC) therapy may represent a new alternative solution in FIA, as it is able to inhibit the inflammation and promote the regeneration process at the same time [10]. MSCs represent a heterogeneous group of multipotent, fibroblast-like, plastic-adherent cells which have the ability of self-renewal and potential to differentiate into a variety of epithelial and mesenchymal cell type like chondroblasts, osteoclasts, or adipocytes [10]. Human MSCs have no major histocompatibility complex (MHC) class II or costimulatory molecule expression (CD40, CD80, and CD86), and the level of HLA class I antigen is also low [11, 12]. This does not only allow autologous treatment but makes allogeneic MSC treatment also safe and simple to perform [10]. A recurrent young Crohn's disease (CD) patient was treated with autologous ASC transplantation, and the recurrence was not detectable for the next three months of follow-up [13]. In a phase I clinical and phase II trial using the adipose mesenchymal stem cell (ADSC) transplantation therapy, the rate of complete fistula closure at the end of week 8 was 75% [14] and 70.8% [15]. A phase III clinical trial was performed by ADSC transplantation for complex perianal fistulas in patients with CD, and the results showed that ADSC transplantation is an effective and safe treatment method and the rate of combined remission did not change over the one-year follow-up (58/103 (56.3%)) [16]. Although more and more reports have been reported on ADSC therapy for complex anal fistula, few reports have been reported in FIA, and animal models are an effective evaluation method for curative effect.

In this study, we established a FIA model of Japanese rabbit and evaluated the effect of four treatments on fistula healing: ADSC transplantation, acellular small intestinal submucosa (ASIS), seton, and PBS as negative control. High-throughput RNA sequencing was also performed to investigate the anal tissue (normal and ADSC treatment group) expression profile of mRNA.

## 2. Materials and Methods

### 2.1. Cell Lines and Culture

Rabbit ADMSCs were purchased from Procell Life Science & Technology Co. Ltd. (Wuhan, China). Cells were successfully cultured in a DMEM-F12 medium which is supplemented with 10% fetal bovine serum (FBS) and penicillin/streptomycin (Gibco, NY, USA) in a humidified incubator at 37°C with 5% CO_2_ and 95% air as well.

### 2.2. Establishing the Rabbit FIA Model

The animal use protocol has been reviewed and approved by the Institutional Animal Care and Use Committee of the Jining Medical University (the animal ethics committee of Jining Medical University Affiliated Hospital (approval number: 2019-FY-012)). The rabbit FIA model was herein established under general anesthesia. A temperature-controlled electric knife (150-450°C) was used to make an anal fistula between 0.5 and 1 cm from the perianal edge of the rabbit and to penetrate it between 0.5 and 0.7 cm from the anal edge of the rabbit under the guidance of anal endoscopy. The rubber band (*d* = 0.2 cm) was suspended in the fistula, and the broken ends of the rubber band were ligated with silk thread to ensure that the floating rubber band was movable in the fistula. All rabbits have antibite devices to prevent biting the rubber bands and licking the inside and outside of the fistula. And 26 days later, the central fistula and peripheral granulation tissue were similar to the clinical features of anal fistula. Finally, 20 models were successfully established; no anal fistula healing was observed within 60 days.

### 2.3. Design and Treatment

Five untreated rabbits were used as the normal control group without any treatment. The 20 FIA rabbits were randomly divided into the cell-treated group (FIA-ADMSCs), the seton-treated group (FIA-seton), the ASIS-treated group (FIA-ASIS), and the control group (FIA-PBS), five rabbits per group.

FIA-ADMSCs and FIA-PBS: the rabbits were given injections of 2 × 10^6^ ADMSCs or equal volume of phosphate-buffered saline (PBS) per fistula, respectively. FIA-seton: the rubber band was inserted into the anal fistula of rabbits with a probe and perforated from the outside to the inside of the fistula. Tighten the rubber band at both ends and ligate the basement with silk thread. After two weeks, the rubber band falls off and the fistula heals. FIA-ASIS: fill the fistula with the ASIS (Shaanxi Ruisheng Biotechnology Co. Ltd., China) of the same appropriate size. All fistulas have undergone curettage and suturing of the internal opening before injection: we used a self-made special anus fistula planer to remove the proliferation tissue around the fistula wall and the internal opening, then sutured the internal opening with 3-0 absorbable suture under the guidance of a nasoscope. There was no drainage in fistula after treatment. The healing standard of anal fistula in the rabbit model was that the external wound healed naturally and there was no secretion around the anus.

Rabbits were sacrificed 70 days after ADMSC transplantation. At the end of experiments, the anal tissues (normal, FIA-PBS, FIA-seton, FIA-ASIS, and FIA-ADMSCs) were fixed in 4% saline-buffered formalin.

### 2.4. RNA-seq

Total RNA was processed by RNA enrichment. PolyA tail was enriched by magnetic beads with OligodT. The obtained RNA was fragmented by interrupting buffer and retranscribed by random N6 primers. Then, the two-stranded DNA was synthesized to form double-stranded DNA. The synthesized double-stranded DNA was flattened and phosphorylated at the 5′ end. The 3′ end formed a sticky end protruding an “A” and then connected to a bubbly junction with a protruding “T” at the 3′ end. The conjugates were amplified by PCR with specific primers. The products of PCR were thermally denatured into single-stranded DNA, and then the single-stranded cyclic DNA library was obtained by cyclization of single-stranded DNA with a bridge primer. Then, online sequencing was performed. The source and reference Genome Version of the oryctolagus_cuniculus for sequencing is GCF_000003625.3_OryCun2.0_ncbi_20190314.

### 2.5. Statistical Analysis

The Student *t*-test was used to analyze the differences available in quantitative variables between the mentioned groups by using the SPSS 16.0 software (SPSS, IL, USA). Herein, *P* < 0.05 was considered statistically significant.

## 3. Results

### 3.1. Fistula Healing Time of ADSC Transplantation Is the Shortest

**Figure 1:**
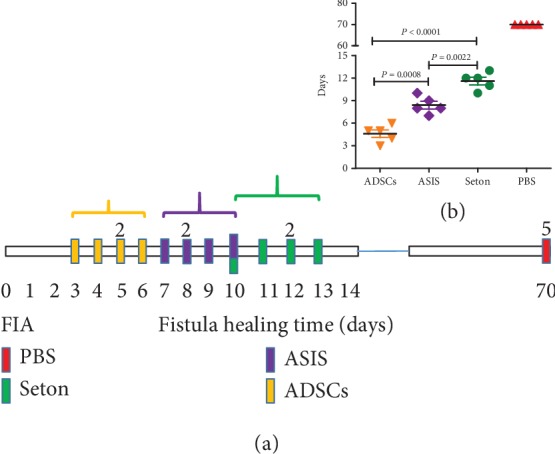
Fistula healing time. (a, b) Patterns show the healing time of each fistula of the four treatments. FIA: fistula-in-ano; PBS: phosphate-buffered saline; ASIS: acellular small intestinal submucosa; ADSCs: adipose-derived stem cells.

All rabbits were treated at the same time (day 0); the healing of fistula was observed every day. The ADSC transplantation group fistulas were healing between 3 and 6 days, the ASIS group between 7 and 10 days, the Seton group between 10 and 13 days, and no fistula healing was observed for 70 days by PBS treatment (Figure 1(a)). Statistical differences of healing time between different groups were analyzed. ADSC treatment has the shortest time to heal the fistulas compared to the ASIS group (*P* = 0.0008) and the seton group (*P* < 0.0001), and ASIS is shorter than the seton group (*P* = 0.0022) (Figure 1(b)).

### 3.2. Anal Healing Evaluation after Treatment

**Figure 2:**
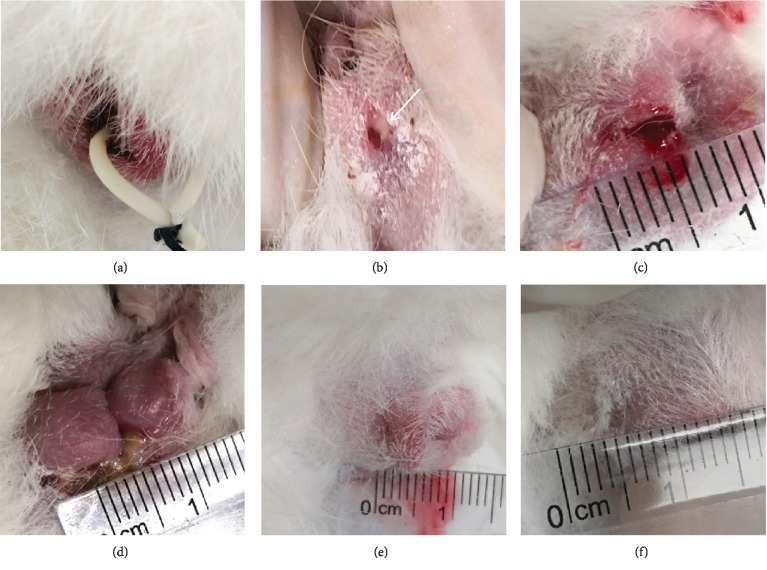
The effect of different treatments on fistula. (a) The FIA model was established by a rubber band. (b) Fistula formation after the removal of the rubber band. (c) PBS injection and the fistulas were not healing, the fluid constantly overflowed from the fistula. (d) Seton cutting treatment, the fistula was healing, but there were anal deformation and scar formation. (e) ASIS promoted fistula healing. (f) ADSC transplantation promoted fistula healing without an obvious scar.

The rabbit FIA model was successfully constructed (Figure 2(a)), and four treatments were implemented after curettage and suturing of the internal opening. As a control group, no fistula healing was observed at the end of the experiment by PBS treatment, and the fluid is constantly flowing out of the fistula (Figure 2(c)). Seton treatment is a well-known traditional operation for FIA. In this study, the fistulas heal eventually, but the shape of the anus is deformed, serious defect marks at the seton cutting site are formed, and the aesthetics is obviously reduced (Figure 2(d)). ASIS treatment healed the fistulas with no significant scar left and no change in the shape of the anus (Figure 2(e)). The ADSC transplantation healing effect is better than that of ASIS, and the anus is similar to that of normal rabbits (Figure 2(e)).

### 3.3. Histopathology Evaluation after Treatment

**Figure 3:**
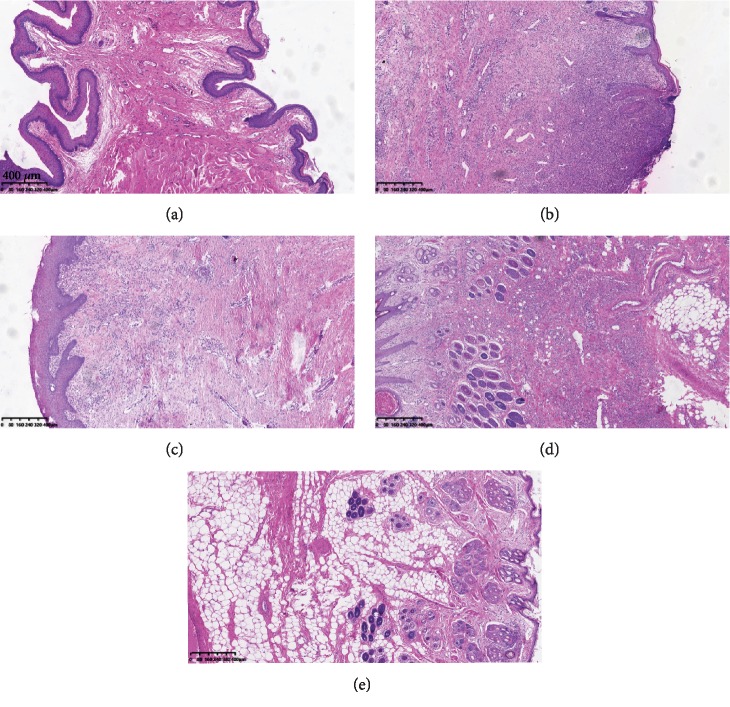
Pathological histology of fistula in different treatments: (a) normal anal tissue, (b) PBS injection, (c) seton cutting treatment, (d) ASIS treatment, and (e) ADSC transplantation.

Normal anal tissue was enriched in fibrous connective tissue under the stratified squamous epithelium (Figure 3(a)). After PBS injection, the fistulas did not heal and were enriched in inflammatory cells (Figure 3(b)). In seton cutting treatment, the fistulas were healing, but the scar is obvious, and the anal tissue was enriched in granulation tissue (Figure 3(c)). In ASIS treatment, the anal tissue was enriched in new-born tissue, the granulation tissue was reduced, adipose tissue was increased, and new hair follicle glands appeared. In ADSC transplantation, the anal tissue was enriched in adipose tissue and new hair follicle glands, there were no granulation and inflammatory cells.

### 3.4. RNA-seq Analysis

**Figure 4:**
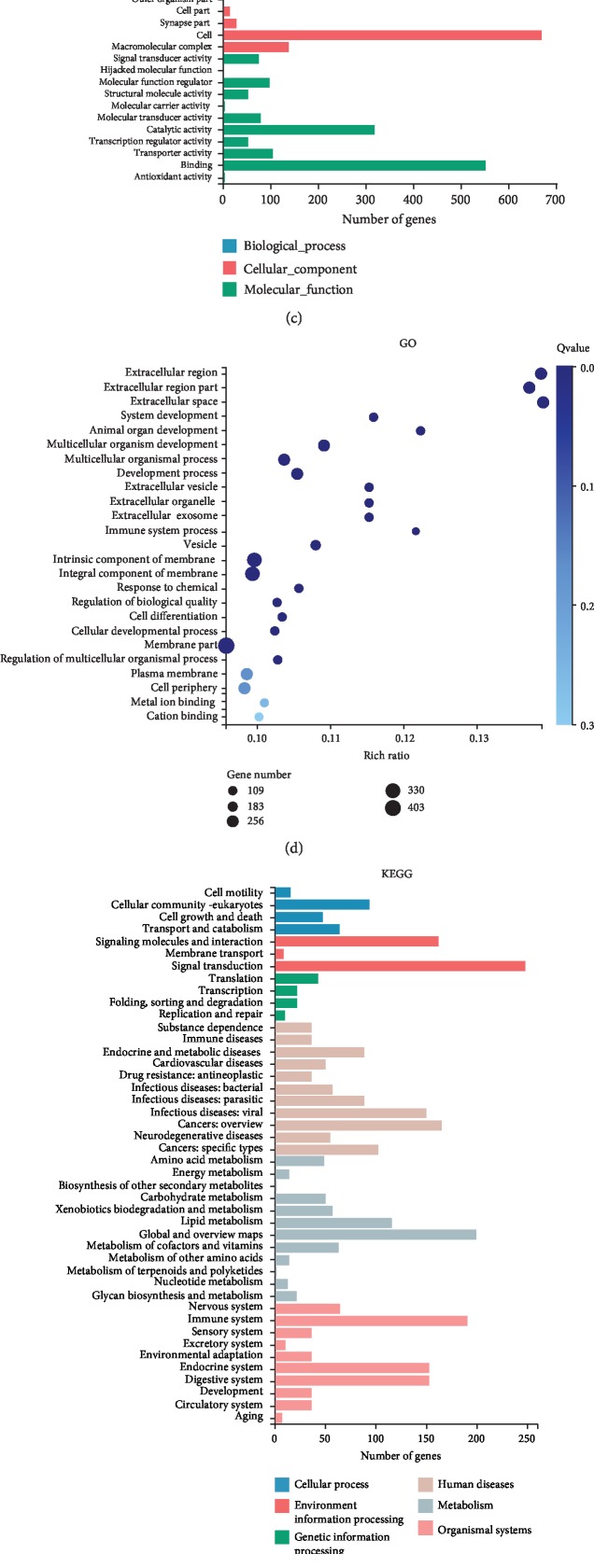
RNA-seq analysis. (a) Differential expression genes in volcanic maps. (b) Differential gene clustering. (c) Gene Ontology classification. (d) Gene Ontology enrichment analysis. (e) KEGG classification. (f) KEGG enrichment analysis.

**Figure 5:**
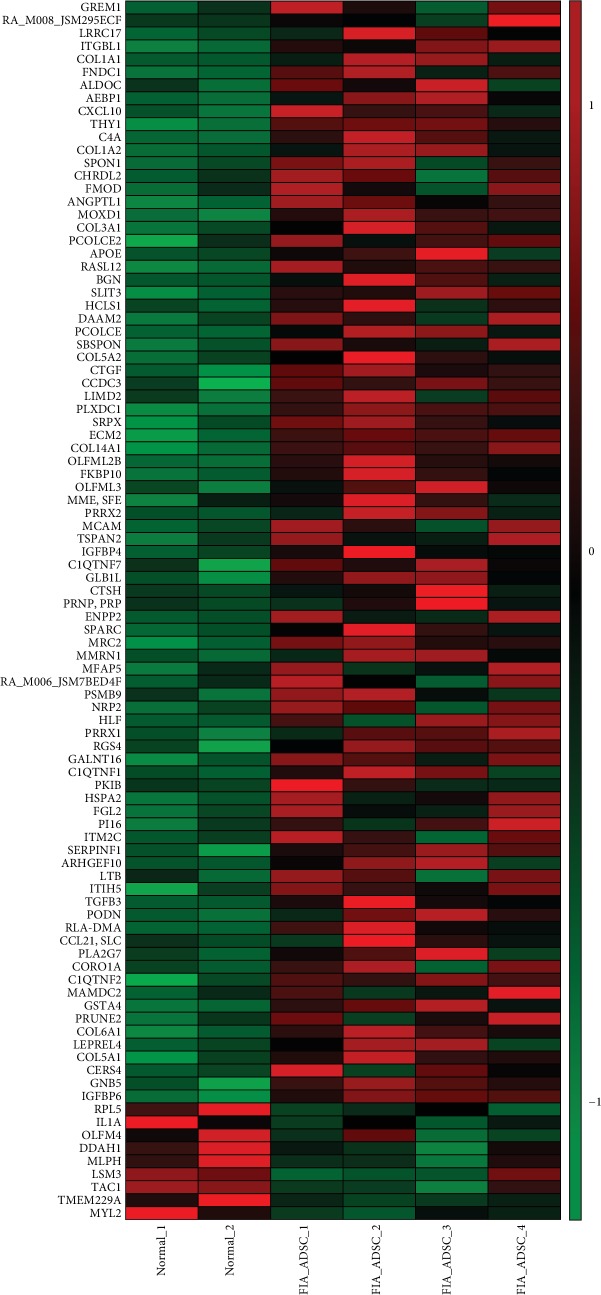
Thermographic analysis of differentially expressed genes in normal and ADSC transplantation fistulas. mRNA expression levels of two normal anal tissues and 4 healing fistulas after ADSC transplantation, each row represents an individual gene, and colors correspond to differential expression variance: green indicates lower levels while red depicts higher levels.

Four ADSC-treated healing rabbit anal tissues and two normal corresponding position anal tissues were used for RNA-seq detection. Among 1747 differentially expressed genes, 1361 were upregulated and 386 were downregulated (fold change (linear)≥±2, *P* < 0.0001) (Figure 3(a)). However, the clustering results showed that the ADSC treatment group and the normal control group could not be significantly separated (Figure 3(b)). It is suggested that the type of tissue after fistula healing by ADSC transplantation closes to normal anal tissue. GO analysis showed that the differentially expressed genes were mainly involved in system development and multicellular organism development (Figure 3(d)). KEGG enrichment analysis showed that differential genes are mainly enriched in signal transduction, immune system, signaling molecules and interaction, etc. (Figure 4(e)). The KEGG pathway analysis results showed that differential genes are significantly enriched in pathways of metabolism of xenobiotics by cytochrome P450, drug metabolism-other enzymes, cytokine-cytokine receptor interaction, protein digestion and absorption, PI3K-Akt signaling pathway, etc. (Figure 4(f)).  Figure 5 demonstrates some differentially significant molecules (85 up and 9 down) that may play an important role in stem cell therapy for fistula healing.

## 4. Discussion

In this study, our data showed that ADSC transplantation is an effective treatment for fistula healing, which have the shortest healing time and fistula filled with adipocytes without scar and inflammation. Rat [17], dog [18], and pig models were also established even the complex FIA [19], but no mouse model was established up to now. Rabbits are a good animal model for fistula research, which has been used to establish a model of transsphincteric fistula [20], aortocavitary fistula [21], esophageal anastomotic fistula [22], urinary fistula [23], and perianal fistula [24]. Rabbits have suitable body size, are easy to obtain, and can establish a large number of FIA models at a time; using this model can improve the therapeutic schedule.

Prolonged placement of noncutting setons remains a “gold standard” in the palliative treatment of fistulas [25]. Sphincter-saving operations should be preceded by prolonged drainage with a noncutting seton until inflammation within the fistulous track recedes [26]. Cutting setons are not recommended due to the risk of damage to the sphincter or deformation of the anus [25]. In our study, the rabbit fistulas are healing at the last time after noncutting setons, but the anal deformation is serious, and fistula has a long healing time with scar and inflammation left. This is similar to the effect of human patient treatment with noncutting setons, which causes great pain to patients.

The anal fistula plug, derived from porcine small intestinal submucosa (SIS), is a strong pliable tissue denuded of cells that provides a scaffold for host fibroblasts to promote tissue healing and repair damaged tissue [27]. SIS is safe but modestly effective in long-term follow-up, with success rates varying from 24% to 88%; the failure rate may be due to its extrusion from the fistula tract [28]. In our study, ASIS treatment healed the rabbit fistulas with no significant scar left and no change in the shape of the anus. Pathologic results showed that the healing fistula was enriched in new-born tissue and the granulation tissue was reduced, adipose tissue was increased, and new hair follicle glands appeared. Despite the excellent therapeutic effect of ASIS, the healing time was still lower than that of the ADSC transplantation group. In addition, not all patients will choose this treatment because of the high cost.

Stem cell therapy is the most promising treatment; at present, several stem cell treatments have shown good results, which may be a prospect of treatment. Human MSCs have no MHCII or costimulatory molecule expression, and the level of HLA class I antigen is also low [11, 12]. No obvious immune rejection response was found in our FIA model after rabbit ADMSC transplantation. Although it is no controversy for FIA patients to transplant their own ADMSCs at present [5], the allogeneic expanded adipose-derived stem cells (Cx601) have been used even in a phase III clinical trial [16, 29]. The results suggest that human ADMSCs can be produced in batches based on its immune exemption.

Recently, research of ADMSC treatment for anal fistula mainly focuses on the therapeutic evaluation of animal and human experiments, but there is hardly any research on the molecular mechanism. In this study, we carried out RNA-seq analysis and obtained many differentially expressed genes. GO analysis showed that the differentially expressed genes were mainly involved in system development and multicellular organism development, and our pathological results also confirm this result. KEGG enrichment analysis showed that differential genes are mainly enriched in the immune system, as shown in thermal maps like GREM1, TGFB3, CXCL10, IL1A, CCL21, and C4A. Furthermore, collagen protein expression levels were all increased, like COL1A1, COL1A2, COL3A1, COL5A1, COL5A2, COL6A1, and COL14A1, which play an important role in fistula healing. We will reveal the role of these molecules in fistula healing in future work.

Although ADMSC treatment for anal fistula has been in the clinical stage and shows good therapeutic effect, there are still many problems to be solved: (1) how to improve the rate of combined remission?; (2) how to prepare standard stem cell products?; (3) how to standardize the treatment plan? More follow-up studies are needed to answer these questions but not all research institutions can conduct clinical trials. A large number of rabbit FIA models are necessary to complete these experiments. Future research will gradually solve the problem, and stem cell therapy for anal fistula may become the first mature product in clinical treatment.

## 5. Conclusions

Our findings demonstrate that ADSCs rapidly promote fistula healing through differentiation, a promising stem cell therapy for FIA. The rabbit is an effective animal model for evaluating FIA therapeutic options.

## Data Availability

The data used to support the findings of this study are available from the corresponding authors upon request.

## References

[1] Bhatt S., Jain B. K., Singh V. K. (2017). Multi detector computed tomography fistulography in patients of fistula-in-ano: an imaging collage. *Polish Journal of Radiology*.

[2] Zanotti C., Martinez-Puente C., Pascual I., Pascual M., Herreros D., Garcia-Olmo D. (2007). An assessment of the incidence of fistula-in-ano in four countries of the European Union. *International Journal of Colorectal Disease*.

[3] Sugrue J., Mantilla N., Abcarian A. (2017). Sphincter-sparing anal fistula repair: are we getting better?. *Diseases of the Colon and Rectum*.

[4] Sainio P. (1984). Fistula-in-ano in a defined population. Incidence and epidemiological aspects. *Annales Chirurgiae et Gynaecologiae*.

[5] Lobascio P., Balducci G., Minafra M. (2018). Adipose-derived stem cells (MYSTEM^®^ EVO Technology) as a treatment for complex transsphincteric anal fistula. *Techniques in Coloproctology*.

[6] Williams J. G., MacLeod C. A., Rothenberger D. A., Goldberg S. M. (1991). Seton treatment of high anal fistulae. *The British Journal of Surgery*.

[7] Lin H., Jin Z., Zhu Y., Diao M., Hu W. (2019). Anal fistula plug vs rectal advancement flap for the treatment of complex cryptoglandular anal fistulas: a systematic review and meta-analysis of studies with long-term follow-up. *Colorectal Disease*.

[8] Schwandner O., Stadler F., Dietl O., Wirsching R. P., Fuerst A. (2008). Initial experience on efficacy in closure of cryptoglandular and Crohn’s transsphincteric fistulas by the use of the anal fistula plug. *International Journal of Colorectal Disease*.

[9] O'Connor L., Champagne B. J., Ferguson M. A., Orangio G. R., Schertzer M. E., Armstrong D. N. (2006). Efficacy of anal fistula plug in closure of Crohn’s anorectal fistulas. *Diseases of the Colon and Rectum*.

[10] Bor R., Fabian A., Farkas K., Molnar T., Szepes Z. (2018). Human mesenchymal stem cell therapy in the management of luminal and perianal fistulizing Crohn’s disease - review of pathomechanism and existing clinical data. *Expert Opinion on Biological Therapy*.

[11] Klyushnenkova E., Mosca J. D., Zernetkina V. (2005). T cell responses to allogeneic human mesenchymal stem cells: immunogenicity, tolerance, and suppression. *Journal of Biomedical Science*.

[12] Le Blanc K., Tammik C., Rosendahl K., Zetterberg E., Ringden O. (2003). HLA expression and immunologic properties of differentiated and undifferentiated mesenchymal stem cells. *Experimental Hematology*.

[13] García-Olmo D., García-Arranz M., García L. G. (2003). Autologous stem cell transplantation for treatment of rectovaginal fistula in perianal Crohn’s disease: a new cell-based therapy. *International Journal of Colorectal Disease*.

[14] Garcia-Olmo D., Garcia-Arranz M., Herreros D., Pascual I., Peiro C., Rodriguez-Montes J. A. (2005). A phase I clinical trial of the treatment of Crohn’s fistula by adipose mesenchymal stem cell transplantation. *Diseases of the Colon and Rectum*.

[15] Garcia-Olmo D., Herreros D., Pascual I. (2009). Expanded adipose-derived stem cells for the treatment of complex perianal fistula: a phase II clinical trial. *Diseases of the Colon and Rectum*.

[16] Panés J., García-Olmo D., van Assche G. (2016). Expanded allogeneic adipose-derived mesenchymal stem cells (Cx601) for complex perianal fistulas in Crohn’s disease: a phase 3 randomised, double-blind controlled trial. *Lancet*.

[17] Guo X. T., Dong Q. J., Cao Y. Q. (2009). Effects of mild moxibustion on angiogenesis and microcirculation in wound repair after operation of anal fistula in rats. *Zhong Xi Yi Jie He Xue Bao*.

[18] Lombardi R. L., Marino D. J. (2008). Long-term evaluation of canine perianal fistula disease treated with exclusive fish and potato diet and surgical excision. *Journal of the American Animal Hospital Association*.

[19] Ba-Bai-Ke-Re M.-M.-T.-J. A., Chen H., Liu X., Wang Y.-H. (2017). Experimental porcine model of complex fistula-in-ano. *World Journal of Gastroenterology*.

[20] Benlice C., Yildiz M., Baghaki S. (2016). Fistula tract curettage and the use of biological dermal plugs improve high transsphincteric fistula healing in an animal model. *International Journal of Colorectal Disease*.

[21] Martel-Arquette A., Tjostheim S. S., Miller J., Carlson J., Mans C. (2019). Aortocavitary fistula secondary to vegetative endocarditis in a rabbit. *Journal of Veterinary Cardiology*.

[22] Xue X., Li C., Yan Y. (2015). Autografting mesenchymal stem cells with fibrin sealant for the therapy of esophageal anastomotic fistula. *Zhonghua Yi Xue Za Zhi*.

[23] GTDS M., Albuquerque A. V., Martins Filho E. D. (2018). Bacterial cellulose to reinforce urethrovesical anastomosis. A translational study. *Acta Cirúrgica Brasileira*.

[24] Rafati M., Hosseini S. V., Moradian F., Zamani M., Khazraei H., Mokhtari M. (2018). Human amniotic membrane effect on perianal fistula healing in rabbits: an experimental study. *Iranian Journal of Medical Sciences*.

[25] Hermann J., Eder P., Banasiewicz T., Matysiak K., Lykowska-Szuber L. (2015). Current management of anal fistulas in Crohn’s disease. *Gastroenterology Review*.

[26] Michelassi F., Melis M., Rubin M., Hurst R. D. (2000). Surgical treatment of anorectal complications in Crohn’s disease. *Surgery*.

[27] Johnson E. K., Gaw J. U., Armstrong D. N. (2006). Efficacy of anal fistula plug vs. fibrin glue in closure of anorectal fistulas. *Diseases of the Colon and Rectum*.

[28] Limura E., Giordano P. (2015). Modern management of anal fistula. *World Journal of Gastroenterology*.

[29] Panés J., García-Olmo D., van Assche G. (2018). Long-term efficacy and safety of stem cell therapy (Cx601) for complex perianal fistulas in patients with Crohn’s disease. *Gastroenterology*.

